# Pet feeding habits and the microbiological contamination of dog food bowls: effect of feed type, cleaning method and bowl material

**DOI:** 10.1186/s12917-023-03823-w

**Published:** 2023-12-07

**Authors:** Federica Raspa, Achille Schiavone, Daniele Pattono, Davide Galaverna, Damiano Cavallini, Marica Vinassa, Domenico Bergero, Alessandra Dalmasso, Maria Teresa Bottero, Emanuela Valle

**Affiliations:** 1 Department of Veterinary Sciences, University of Turin, Grugliasco, 10095 Italy; 2 Department of Veterinary Sciences, University of Bologna, 40064 Ozzano Dell’Emilia, Bologna, Italy; 3 About Petfood Di Davide Galaverna, Vinovo, 10048 Italy

**Keywords:** One Health principle, Pets, Nutrition, Food bowl, Microbiological contamination

## Abstract

**Background:**

Safe pet feeding practices and food bowl hygiene measures are important for minimising the risk of microbiological contaminations in the domestic environment. This study compares the practices reported by dog and cat caregivers, and investigates whether cleaning method, feed type or bowl material affects the microbiological contamination of dog food bowls.

**Results:**

Data from 351 dog caregivers and 186 cat caregivers were collected via an online survey. The majority of dogs (70.7%) were fed twice daily, whereas cats (43%) were mostly fed ad libitum. The most common material for dog food bowls was metal (67.1%) versus plastic (38.1%) and metal (37.6%) for cats. Dog food bowls were most frequently cleaned after each meal (35.7%); whereas for cats, 21.5% were cleaned after each meal, 22.7% once a day and 19.3% 2–3 times a week. Total mesophilic aerobic bacteria counts (TMABc), *Enterobacteriaceae* counts and pathogenic bacteria (*Salmonella* spp., *Campylobacter* spp., Verotoxigenic *E. coli* [VTEC]) were assessed for 96 dog food bowls. TMABc were higher in metal *vs*. plastic bowls (*p* < 0.001) and in those used for wet food *vs*. dry food (*p* = 0.0397). *Enterobacteriaceae* counts were higher in bowls washed by hand *vs*. dishwasher (*p* = 0.0515), whereas no differences were found between hand washing *vs*. dry wiping. *Salmonella* spp., *Campylobacter* spp. or *E. coli* VTEC contaminations were not detected.

**Conclusions:**

The surveyed Italian dog and cat caregivers reported different habits concerning feeding frequency, food bowl material and cleaning frequency. Wet food and metal bowls were associated with higher levels of microbiological contamination of dog food bowls. Furthermore, in relation to wet washing methods, contaminations were likely to be greater following hand washing than they were following the use of a dishwasher. Practical guidelines for safe feeding practices and hygiene measures are needed to minimise the risk of microbiological contaminations in domestic environments.

## Background

Guidelines for safe pet feeding practices and food bowl hygiene measures are not presently available for pet caregivers [1]. High risk pet feeding practices such as the provision of raw meat or other uncooked ingredients and poor food and water bowl hygiene measures could have adverse health consequences for both pets and humans. This aspect is particularly important in view of the “One Health” principle which underscores the reciprocal health cycle between humans, animals and the environment. In fact, high-risk pet feeding practices and poor food and water bowl hygiene can create the conditions for microbial contamination of domestic environments, including those where human food is prepared and stored [2]. Contaminated food in the kitchen can lead to cross-contamination of household equipment (sponges, knives and spoons) and surfaces and the hands of the people managing the pet food [3, 4]. Pet food bowls can also act as a vehicle for bacterial transmission [1]. Scott et al. [5] reported the surface of dog food bowls to have the ninth highest level of microbial contamination (median total mesophilic aerobic bacteria counts, TMABc 3.9 × 10^2^ cm^2^) of the household objects used on a daily basis. Donofrio et al. [6] found pet toys and pet water dishes to have the highest bacterial counts (median TMABc ranging between 10^2^ and 10^3^ cm^2^), making pet-related items a potential source of microbiological contamination. The main concerns are related to the safe handling and use of raw meat ingredients since raw meat diets for pets have been recognised as a source of zoonotic bacterial pathogens and thus as a source of hazards for both pets and people [2]. The scientific literature addressing the hygiene of raw diets reports the occurrence of positive testing to zoonotic bacterial pathogens within the home. For example, van Bree et colleagues [7] isolated *Escherichia coli* serotype O157:H7 from 23% of the 35 commercial frozen raw meat-based diets for companion animals they analysed, as well as *Listeria monocytogenes* from 43% and *Salmonella* spp. from 20% of these commercial diets. Moreover, recent findings have associated the risk of human exposure to bacteria as a consequence of contact with contaminated dry dog foods [6]. In particular, Weese et al. [8] isolated *Clostridium difficile* from 6 out of 84 dog food bowls. Moreover, both high-moisture and dry pet foods have been reported to be a potential source of *Salmonella* spp. infection, and the practice of re-wetting remaining pet food left in food bowl has been reported to encourage bacterial growth [9, 10].

Different materials are used for manufacturing pet food bowls, including plastic, metal (stainless steel or similar) and ceramic. These materials are subject to contamination by microorganisms, and biofilm formation could be influenced by various factors, including food bowl surface characteristics (i.e. whether they are smooth, rough or irregularly shaped), their condition (i.e. before *vs.* after the cleaning process, new *vs.* old) and environment characteristics (i.e. temperature, the presence of food remnants) [11, 12].

At present, very little literature exists concerning the habits of pet caregivers related to pet feeding and food bowl hygiene practices. Moreover, as stated in a recent study [1], it is particularly important that our understanding regarding the microbiological contamination of dog food bowls in terms of the total mesophilic aerobic bacteria counts (TMABc) as well as the specific pathogenic bacteria involved is addressed. Thus, the aims of the present study were: (1) to obtain data on the pet feeding practices employed and the food bowl hygiene measures adopted by Italian dog and cat caregivers; and (2) to analyse the microbiological contamination – in terms of total mesophilic aerobic bacteria counts (TMABc), *Enterobacteriaceae* counts and pathogenic bacteria (*Salmonella* spp., *Campylobacter* spp., Verotoxigenic *E. coli* [VTEC]) – of dog food bowls according to the cleaning method (CM), feed type (FT) and bowl material (BM). The authors hypothesized that differences may exist in the pet feeding practices and the feed bowl hygiene measures adopted by dog and cat caregivers, and that the use of different cleaning methods, feed types and bowl materials could significantly influence the microbiological contamination of dog food bowls.

## Results

### The survey

The open survey was shared by word of mouth and on social media (Facebook©) for 24 weeks. All caregivers of dogs and cats were free to fill in the questionnaire on a voluntary basis. A total of 351 Italian dog caregivers and 186 cat caregivers filled out the online questionnaire.

The socio-demographic characteristics of the surveyed pet caregivers are reported in Table 1; pet characteristics are reported in Table 2; and the feeding and bowl characteristics used are summarised in Table 3. According to Table 1, 83.2% of respondents were women and around 50% were aged less than 35 years. Of the total sample, 59.1% owned dogs and almost all respondents considered their pet a member of the family.

**Table 1:** Socio-demographic characteristics of the surveyed pet caregivers

Characteristics	*n* of valid responses, %
**Gender**	***n* = 430, 100%**
Woman	*n *= 358, 83.2%
Man	*n *= 69, 16%
Not specified	*n *= 3, 0.7%
**Age**	***n *= 430, 100%**
< 18 years	*n *= 5, 1.2%
18–25 years	*n *= 124, 28.8%
25–34 years	*n *= 132, 30.7%
35–44 years	*n *= 71, 16.5%
45–54 years	*n *= 57, 13.3%
55–64 years	*n *= 32, 7.4%
> 65 years	*n *= 9, 2.1%
**Occupation related to animals**	***n *= 430, 100%**
No	*n *= 232, 54%
Yes	*n *= 198, 46%
**Animal(s) owned (dogs and/or cats)**	***n *= 430, 100%**
Dogs	*n *= 254, 59.1%
Cats	*n *= 80, 18.6%
Dogs and cats	*n *= 96, 22.3%
**Relationship with pet**	***n *= 430, 100%**
Pet	*n *= 37, 8.5%
Work animal	*n *= 2, 0.5%
Friend	*n *= 13, 3.1%
Family member	*n *= 378, 87.9%

**Table 2:** Pet characteristics of the surveyed population

Pet characteristics	Dogs *n* of valid responses, %	Cats *n* of valid responses, %
**Breed**	***n *= 351, 100%**	***n *= 186, 100%**
	*n *= 128, 36.5% mixed breed	*n *= 164, 88.2% European breeds
	*n *= 223, 63.5% pure breed	*n *= 22, 11.8% other breeds
**Sex**	***n *= 351, 100%**	***n *= 186, 100%**
Intact male	*n *= 117, 33.3%	*n *= 7, 3.8%
Intact female	*n *= 70, 19.9%	*n *= 13, 7%
Spayed male	*n *= 49, 14%	*n *= 76, 40.9%
Spayed female	*n *= 115, 32.8%	*n *= 90, 48.4%
**Size**	***n *= 351, 100%**	**n.a**
Small (< 10 kg)	*n *= 71, 20.2%	n.a
Medium (10–25 kg)	*n *= 151, 43%	n.a
Large (25–45 kg)	*n *= 121, 34.5%	n.a
Giant (> 45 kg)	*n *= 8, 2.3%	n.a
**Housing**	***n *= 351, 100%**	***n *= 186, 100%**
Home	*n *= 207, 59%	*n *= 113, 60.8%
Garden/courtyard	*n *= 21, 6%	*n *= 14, 7.5%
Both	*n *= 123, 35%	*n *= 59, 31.7%

**Table 3:** Feeding practices and food bowl hygiene measures employed by the surveyed population

Feeding and bowl characteristics	Dogs *n* of valid responses, %n of valid responses, %	Cats *n* valid responses, %
**Feed type**	***n *= 351, 100%**	***n *= 186, 100%**
Dry and wet commercial pet food	*n *= 183, 52.1%	*n *= 159, 85.4%
Cooked home-prepared diet	*n *= 26, 7.4%	*n *= 5, 2.6%
Raw commercial and home-prepared diet	*n *= 62, 17.7%	*n *= 10, 5.8%
A mix of the above diet types	*n *= 80, 22.8%	*n *= 12, 6.2%
**Daily feeding frequency**	***n *= 351, 100%**	***n *= 186, 100%**
1/day	*n *= 28, 8%	*n *= 4, 2.2%
2/day	*n *= 248, 70.7%	*n *= 48, 25.8%
3/day	*n *= 59, 16.8%	*n *= 36, 19.4%
> 4/day	*n *= 2, 0.6%	*n *= 18, 9.7%
Ad libitum	*n *= 14, 4%	*n *= 80, 43%
**Food bowl material**	***n *= 351, 100%**	***n *= 186, 100%**
Metal	*n *= 236, 67.1%	*n *= 70, 37.6%
Plastic	*n *= 68, 19.5%	*n *= 71, 38.1%
Ceramic	*n *= 34, 9.6%	*n *= 19, 10.5%
Other	*n *= 13, 3.8%	*n *= 26, 13.8%
**Food bowl cleaning frequency**	***n *= 351, 100%**	***n *= 186, 100%**
After each meal	*n *= 125, 35.7%	*n *= 40, 21.5%
Once a day	*n *= 59, 16.7%	*n *= 42, 22.7%
2–3 times a week	*n *= 56, 15.9%	*n *= 36, 19.3%
Once a week	*n *= 57, 16.4%	*n *= 31, 16.6%
2–3 times a month	*n *= 23, 6.5%	*n *= 18, 9.4%
Once a month	*n *= 14, 4%	*n *= 12, 6.6%
Less than once a month	*n *= 12, 3.4%	*n *= 6, 3.3%
Never	*n *= 5, 1.4%	*n *= 1, 0.6%
**Cleaning methods**	***n *= 351, 100%**	***n *= 186, 100%**
Not cleaned	*n *= 4, 1.1%	*n *= 2, 1.1%
Dry wiping	*n *= 60, 17.1%	*n *= 22, 11.7%
Rinse by hand	*n *= 78, 22.1%	*n *= 34, 18.2%
Wash with soap and sponge	*n *= 136, 38.6%	*n *= 86, 46.4%
Dishwasher	*n *= 17, 5.0%	*n *= 16, 8.8%
By hand then the dishwasher	*n *= 56, 16.1%	*n *= 26, 13.8%

According to the pet characteristic profiles summarised in Table 2, most dogs were pure breeds, and the majority of dogs were either intact males (32.8%) or spayed females (33.3%). The predominant dog sizes were medium (43%) and large (34.5%), and 59% of all dogs lived exclusively in the house with their caregivers. As regards the cats’ characteristics, most were European breeds (88.2%) and they were predominantly distributed between spayed males (40.9%) and spayed females (48.4%). The majority of the respondents kept their cats inside their homes without outdoor access (60.8%).

According to Table 3, around half the population of surveyed dog caregivers (52.1%) fed their dogs with dry or wet commercial pet food. The daily feeding frequency for dogs was twice a day for 70.7% of the respondents. Metal bowls were predominantly used for dogs (67.1%). Considering the cleaning frequency of dog food bowls, 35.7% of respondents cleaned the bowl after each meal, and the remainder were almost equally distributed between those who cleaned it once a day (16.7%), once a week (16.4%) and 2–3 times a week (15.9%). As regards the methods used for cleaning dog bowls, the majority of the surveyed population washed bowls by means of wet cleaning methods (81.8%), whereas a smaller proportion used dry wiping methods to clean dog feed bowls (17.1%).

Almost all cats were fed with dry or wet commercial pet food (85.4%). Most were fed ad libitum (43%), and about a quarter of the surveyed population fed their cats twice a day. The proportion of cat caregivers using metal *vs.* plastic bowls was very evenly distributed (37.6% *vs.* 38.1%, respectively), as was the distribution of cat caregivers who cleaned bowls after each meal (21.5%), once a day (22.7%) or 2–3 times a week (19.3%). Similar to dog caregivers, the majority of cat caregivers washed food bowls using one of the wet cleaning methods (87.2%), whereas 11.7% used dry wiping methods only.

### Microbiological contamination of dog bowls

**Fig. 1:**
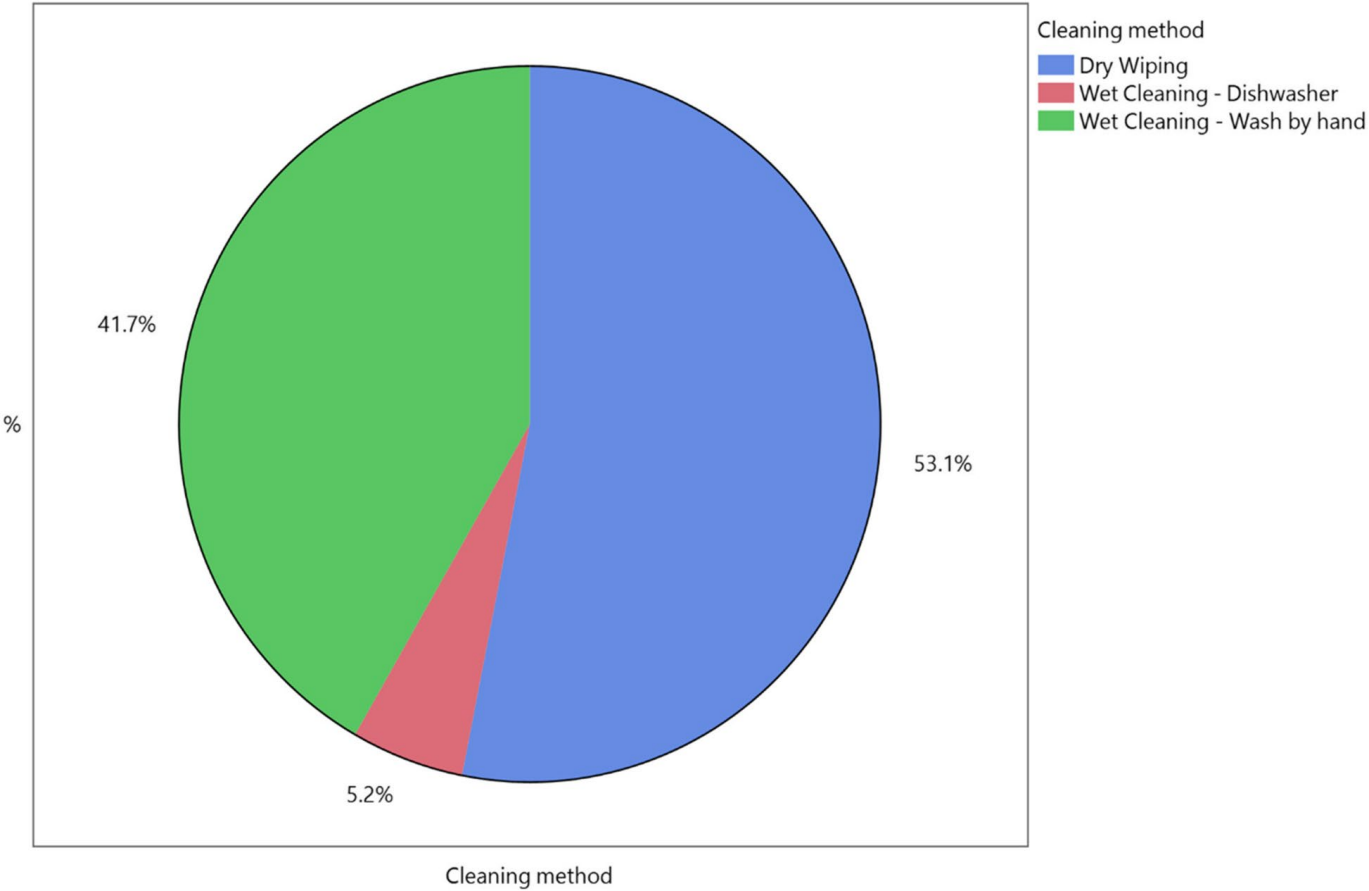
The cleaning methods used by caregivers for the  dog bowls (*n *= 96) subjected to microbiological analysis

**Fig. 2:**
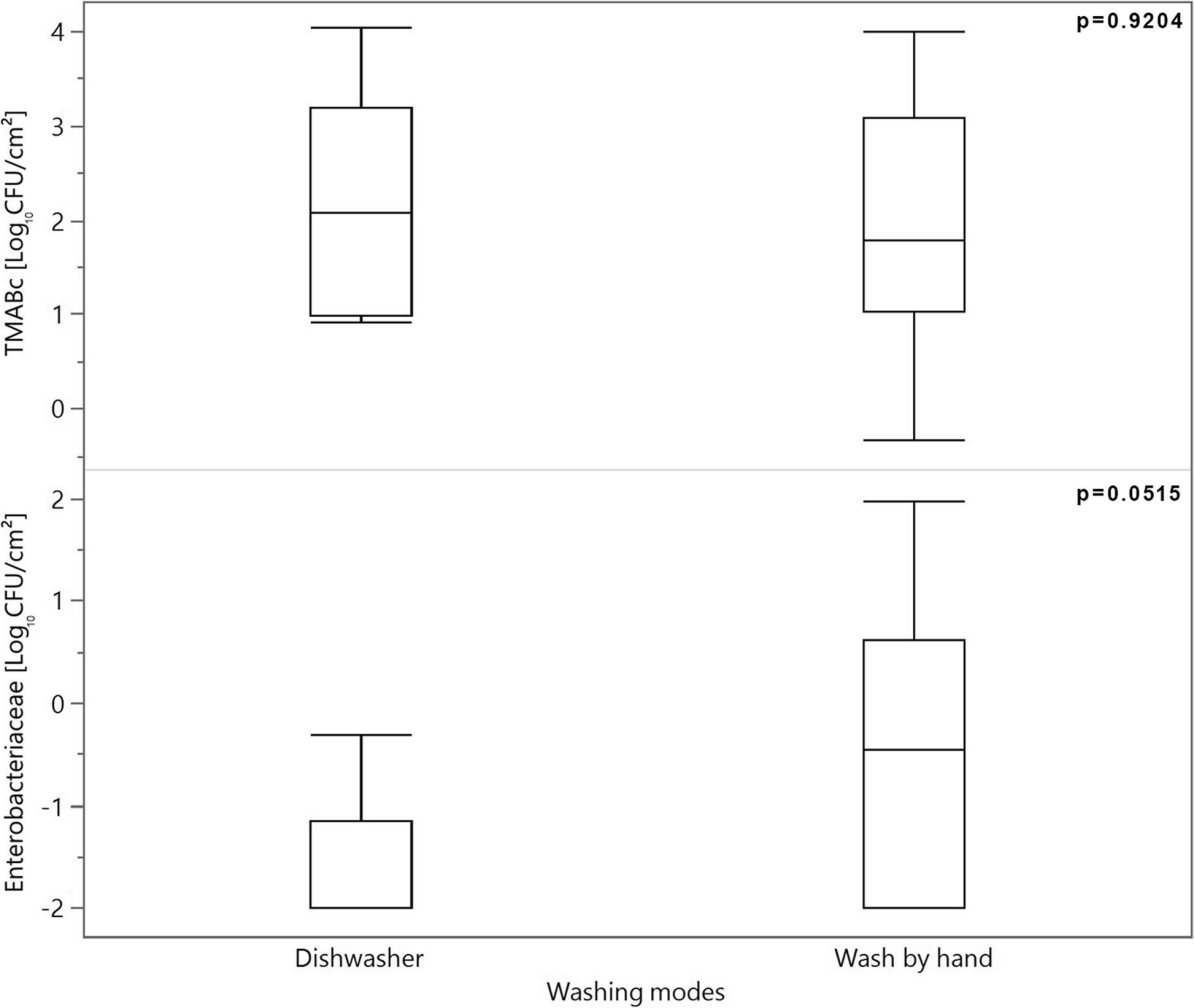
Differences in total mesophilic aerobic bacteria counts (TMABc) and *Enterobacteriaceae* counts in the subsampled dog food bowls (*n *= 96) divided according to the different wet cleaning methods: dishwasher (*n *= 5) *vs*. washing by hand (*n *= 40)

Ninety-six dog caregivers out of the 351 dog caregivers taking part in the questionnaire were willing to participate in the sampling procedure for the microbiological analysis. Therefore, the microbiological contamination of 96 dog bowls was assessed. The distribution of the different types of cleaning methods (CM) adopted by these 96 dog caregivers are shown in Fig. 1: 51 dog caregivers (53.1%) adopted dry wiping, 40 dog caregivers (41.7%) adopted hand washing and 5 dog caregivers (5.2%) used a dishwasher. Exploring the microbiological contamination of dog food bowls according to cleaning methods, no differences were found in TMABc and *Enterobacteriaceae* counts between hand washing and dry wiping. However, as shown in Fig. 2, the *Enterobacteriaceae* counts showed higher values when dog food bowls were washed by hand compared with by dishwasher (*p* = 0.0515). Significantly higher TMABc were observed in dog food bowls in which wet food was provided compared with dry food (*p* = 0.0397; Table 4), whereas no differences were found for *Enterobacteriaceae* counts. Considering bowl material (BM), metal bowls were more significantly contaminated compared with plastic bowls in relation to TMABc (*p* < 0.001, Table 4). No interactions between FT and CM, FT and BM, CM and BM (Table 5) as well as between FT, CM and BM (Table 6) were found.

**Table 4:** Mean (SEM) TMABc (total mesophilic aerobic bacteria counts) and *Enterobacteriaceae* counts expressed as Log_10_ CFU/cm^2^ and *p*-values for comparisons between feed type, cleaning method and bowl material

		***n***	**%**	**TMABc** **[Log**_**10**_**CFU/cm**^**2**^**]**	***p*-value**	**Enterobacteriaceae** **[Log**_**10**_**CFU/cm**^**2**^**]**	***p*-value**
**Feed type**	Dry food	46	51	1.42 (0.19)	0.0397*	-0.77 (0.19)	0.7507
	Wet food	45	49	1.99 (0.19)		-0.66 (0.19)	
**Cleaning method**	Dry wiping	51	56	1.45 (0.18)	0.0672	-0.94 (0.18)	0.1002
	Hand washing	40	44	1.96 (0.20)		-0.49 (0.21)	
**Bowl material**	Plastic	46	51	1.23 (0.19)	< 0.001*	-0.98 (0.20)	0.0594
	Metal	45	49	2.18 (0.19)		-0.45 (0.19)	

^*^Statistical significance: *p* ≤ 0.05

**Table 5:** Mean (SEM) TMABc (total mesophilic aerobic bacteria counts) and *Enterobacteriaceae* counts expressed as Log_10_ CFU/cm^2^ and *p*-values for comparisons between feed type, cleaning method and bowl material pairwise interactions

		**n**	**%**	**TMABc** **[Log**_**10**_**CFU/cm**^**2**^**]**	***p*-value**	**Enterobacteriaceae** **[Log**_**10**_**CFU/cm**^**2**^**]**	***p*-value**
**Food type & cleaning method**	Dry food & dry wiping	26	29	1.02 (0.25)	0.3270	-1.06 (0.25)	0.6331
	Dry food & hand washing	20	22	1.81 (0.29)		-0.48 (0.29)	
	Wet food & dry wiping	25	27	1.87 (0.26)		-0.82 (0.26)	
	Wet food & hand washing	20	22	2.11 (0.29)		-0.50 (0.29)	
**Food type & bowl material**	Dry food & plastic	22	24	0.98 (0.28)	0.7875	-1.10 (0.28)	0.7163
	Dry food & metal	24	26	1.85 (0.26)		-0.44 (0.26)	
	Wet food & plastic	24	26	1.48 (0.26)		-0.87 (0.27)	
	Wet food & metal	21	23	2.49 (0.28)		-0.46 (0.28)	
**Cleaning method & bowl material**	Dry wiping & plastic	25	28	1.23 (0.24)	0.0646	-1.13 (0.24)	0.6094
	Dry wiping & metal	23	25	1.67 (0.27)		-0.74 (0.27)	
	Hand washing & plastic	21	23	1.23 (0.30)		-0.83 (0.31)	
	Hand washing & metal	22	24	2.68 (0.27)		-0.15 (0.28)	

Statistical significance: *p* ≤ 0.05

**Table 6:** Mean (SEM) TMABc (total mesophilic aerobic bacteria counts) and *Enterobacteriaceae* counts expressed as Log_10_ CFU/cm^2^ and *p*-values for comparisons between feed type, cleaning method and bowl material global interactions

		**n**	**%**	**TMABc** **[Log**_**10**_**CFU/cm**^**2**^**]**	***p*-value**	**Enterobacteriaceae** **[Log**_**10**_**CFU/cm**^**2**^**]**	***p*-value**
**Food type & Cleaning method & Bowl material**	Dry food & dry wiping & plastic	14	15	0.85 (0.34)	0.9787	-1.38 (0.35)	0.5442
	Dry food & dry wiping & metal	12	13	1.21 (0.37)		-0.72 (0.37)	
	Dry food & hand washing & plastic	8	9	1.12 (0.45)		-0.76 (0.46)	
	Dry food & hand washing & metal	12	13	2.49 (0.37)		-0.16 (0.37)	
	Wet food & dry wiping & plastic	14	15	1.62 (0.34)		-0.89 (0.35)	
	Wet food & dry wiping & metal	11	12	2.12 (0.39)		-0.77 (0.39)	
	Wet food & hand washing & plastic	10	11	1.34 (0.40)		-0.87 (0.41)	
	Wet food & hand washing & metal	10	11	2.87 (0.41)		-0.14 (0.41)	

Statistical significance: *p* ≤ 0.05

Pathogenic bacteria – *Salmonella* spp., *Camplylobacter* spp. and Verotoxigenic *E. coli* [VTEC] – were not detected in any of the dog bowls analysed.

## Discussion

According to the “One Health” concept, human, animal and environmental health are interrelated. The increasingly close contact between humans and cohabiting pets – considered by 87.9% of the respondents in our study as members of the family – is recognised as contributing toward improving the health of pet caregivers and their families. However, attention needs to be paid to guarantee safe in-home pet feeding practices and the employment of food bowl hygiene measures due to the associated risk of bacterial transmission within the domestic environment [13]. The aim of the present survey carried out on Italian pet caregivers was to study how caregivers managed pet food bowls, with particular attention paid to the frequency with which they cleaned the bowls and the cleaning method adopted. The majority of respondents (52.1% of dog caregivers and 85.4% of cat caregivers) fed their pets commercial pet food (dry or wet commercial pet food), in accordance with the data reported in the scientific literature and the latest official Italian reports [14, 15]. The data obtained by the survey revealed that 35.7% of dog caregivers cleaned their dog’s feed bowls after each meal, 16.7% cleaned it once a day, 16.4% once a week and 15.9% 2–3 times a week. The behaviour of cat caregivers with respect to bowl cleaning was different, with the cleaning frequency more equally distributed between once a day (22.7%), after each meal (21.5%), 2–3 times a week (19.3%) and once a week (16.6%). This may be linked to the fact that many cat caregivers feed their animals ad libitum (43% of the respondents in our survey), and, as such, cat bowls could often be full of feed and thus ad libitum feeding could be related with longer cleaning intervals.

Interestingly, over half of both dog and cat caregivers reported to clean their animal’s bowls by washing with soap and a sponge (38.6% and 46.4%, respectively); the next most frequent cleaning methods were rinsing with water only (22.1% and 18.2%, for dog and cat food bowls respectively), washing by hand and then the dishwasher (16.1% and 13.8%, respectively), and finally by dishwasher only (5% and 8.8%, respectively). The survey carried out by Luisana et al. [1] on 417 dog caregivers from North Carolina revealed different habits. In that study, 36% of dog caregivers cleaned dog bowls with soap and warm water, 33% by dishwasher and 6% with water only. In agreement with the data published by Luisana et al. [1], the majority of dog bowls used by the respondents of the present study were made of metal (67.1%); whereas the proportion of cat caregivers using metal *vs*. plastic bowls was evenly distributed (37.6% *vs.* 38.1%, respectively).

We then evaluated whether the bowl cleaning method adopted by the caregiver (hand washing *vs*. dishwasher and hand washing *vs*. dry wiping), the feed type given (wet food *vs*. dry food) or the bowl material (plastic *vs*. metal) affected the level of microbiological contamination in the dog food bowls. Microbiological analysis was only carried out on dog bowls since the majority of the study respondents were dog caregivers. Indeed, the unbalanced distribution of dog and cat caregivers responding to questionnaire represents a limitation of the present study. The results revealed that the bacterial contamination of dog food bowls was affected by feed type (FT), cleaning method (CM) and bowl material (BM). Regarding FT, wet food was associated with higher TMABc compared with dry food. This could be linked to the fact that higher humidity levels are likely to create more favourable conditions for bacterial growth [11]. Other studies have similarly reported FT to influence the bacterial contamination of dog bowls. For example, Weese et al. [8] found 84 dog bowls used for a raw diet to be 17 times more likely to be contaminated with *Clostridium difficile* than other types of diet. Moreover, several other pathogenic bacteria such as *Salmonella enterica* and *Campylobacter* spp. have been mainly linked to contact with high-moisture foods such as raw meat [16, 17]. That said, recent outbreaks of *Salmonella* spp. in humans were found to be associated with contact with contaminated dry dog foods [6]. In our study, no pathogenic bacteria (*Salmonella* spp., *Campylobacter* spp., and Verotoxigenic *E. coli* [VTEC]) were found in the sampled dog food bowls. However, it is important to underline that bacterial growth is more likely to occur in a situation in which a portion of food is left in the bowl for 6 or 12 h after feeding and the food has been in contact with the animal’s saliva [9]. Of the surveyed dog caregivers, 52.4% cleaned the dog bowl at least once a day (35.7% after each meal and 16.7% once a day), and this could have had an impact on our findings. Accordingly, it could be of interest to perform the same microbiological analyses in cat food bowls considering that 43% of the Italian cat caregivers surveyed replied that they fed their animals ad libitum and cleaned the feed bowls less frequently compared with dog caregivers. Moreover, we found that the cleaning method adopted had an effect on bacterial growth, with higher levels of bacterial growth associated with hand washing with respect to the use of a dishwasher (Fig. 1). However, no differences in microbiological contamination were found between hand washing and dry wiping (Table 4). Luisana and colleagues [1] found that dog bowls washed following a hygiene protocol involving hot water (71.1 °C) and soap after each use had lower mean total mesophilic aerobic bacteria counts compared with dog bowls washed with cold/lukewarm water. The temperature of the water together with the use of sponges for wet cleaning may explain the higher levels of microbial contamination found in our study when the dog food bowls were washed by hand compared with by a dishwasher. Indeed, bacteria have been shown to survive on sponges, cloths and kitchen utensils/equipment after wet cleaning procedures [18]. According to Marotta et al. [19] the sponges or cloths that should exclusively be used for cleaning tools and surfaces related to the kitchen environment are often promiscuously used for other purposes such as the cleaning of pet bowls. This aspect plays an important role in the cross-contamination of household items following the cleaning of animal food/water bowls, and may explain the higher bacterial counts found in bowls washed by hand compared with by a dishwasher. Moreover, an interesting aspect to investigate in the future would be whether caregivers wash dog bowls in the same sink/dishwasher as used for human dishes since cross-contamination may also occur inside the sink/dishwasher.

In our study metal bowls showed higher TMABc counts compared with plastic bowls. This finding is in contrast with the study carried out by Luisana et al. [1], which did not find bowl material to have a significant effect on bacterial counts. However, considering metal bowls, it should be taken into account that the grade of steel, the surface smoothness and the age of the steel promote good cleanability and reduce the risk of corrosion [20, 21]. The geometry of bowls should also be considered in addition to the material as this characteristic can also affect microbial adhesion; smooth surfaces which permit easier cleaning are most desirable from the hygiene perspective [12]. For example, slow-feeding dog bowls are typically designed to include internal ridges or other structures which increase the feeding time, but that make the cleaning process more difficult. Accordingly, we decided to exclude the responses from caregivers who used such bowls in our study, but it will be a further point to explore in the future. The texture of food bowl surfaces should also be considered as well as the age of the bowl as rougher textures and damaged surfaces may be more difficult to clean adequately.

Moreover, since 38.1% of the cat caregivers of the present study reported feeding their cats using plastic bowls, it would be interesting for future research to assess the microbiological contamination of cat bowls since, to the best of our knowledge, no such data are available.

## Conclusions

Our survey reveals Italian dog and cat caregivers to exhibit different habits concerning daily feeding frequency, food bowl material, and bowl cleaning frequency. Our study shows that dog food bowls were at a higher risk of microbiological contamination when used with wet food and made of metal. Moreover, hand washing was associated with higher microbiological contamination than washing using a dishwasher. In contrast, no differences were found between hand washing and dry wiping. These findings underline the need to formulate specific practical guidelines for safe feeding and bowl hygiene practices in order to minimise the risk of microbiological contamination in the domestic environment. Further studies should focus on identifying the ideal cleaning method and bowl material to minimise health consequences in pets, their caregivers (and family members) and the domestic environment in light of the “One Health” perspective.

## Materials and methods

The study procedures were approved by the University of Turin Animal Ethics and Welfare Committee (Prot. N°2183/26/07/21). The present procedures as enrolment onto the survey was on a voluntary basis and the participants provide informed consent to participate in the anonymous data collection as per Regulation (EU) n. 2016/679 of the European Parliament and of the Council of 27 April 2016. All methods were performed in accordance with the relevant guidelines and regulations. Informed consent was obtained from all subjects and/or their legal guardian(s). No animal was used in the study and all information was taken from the caregivers only. The written permission/informed consent to use the owned animal is provided by the each animal owner.

### The survey

An online survey in the Italian language was created using Google Forms© to investigate the habits of dog and cat caregivers on the management and hygiene practices of their pets’ food bowls.

The open survey was shared by word of mouth and on social media (Facebook©) for 24 weeks – from June 2021 to November 2021 – and it was actively promoted to different pet enthusiasts, caregivers and breeder groups. A pilot version of the questionnaire was presented to 30 people prior to conducting the survey in order to ascertain whether it was easy to understand. All caregivers were free to fill in the questionnaire on a voluntary basis, regardless of the type of diet supplied to their pet.

The questions selected for the survey were based on those already available in the scientific literature [1, 14]. Each question was developed with the assistance of veterinary experts in the field of animal nutrition. The survey was divided into three different sections. The first Sect. (5 questions) was designed to profile the socio-demographic characteristics of the surveyed pet caregivers. It asked respondents about their: gender, age, whether their occupation was animal-oriented, the animal they owned (dog or cat) and aspects related to their relationship with their pet. In the second Sect. (4 questions), the pet caregivers were asked about their pets’ characteristics, divided according to dogs *vs*. cats, and gathered information about breed, sex, size and housing. The third Sect. (5 questions) included information on the feeding management and bowl hygiene practices, and considered food type, feeding frequency, bowl material, cleaning frequency and cleaning methods.

### Procedures to assess the microbiological contamination of dog bowls

The criteria for inclusion were: being a dog owner, having a healthy dog, willingness to participate in the study, the use of plastic or metal food bowls and the possibility to deliver bowls to the laboratory for sampling. Moreover, caregivers who used slow-feeding dog bowls were excluded since such bowls are typically designed to include internal ridges or other structures which make the cleaning process more difficult. These inclusion and exclusion criteria were decided after the collection of all the compiled surveys as we obtained a higher percentage of answers from dog caregivers who happened to use primarily plastic or metal bowls.

A trained operator sampled each dog bowl using a sponge moistened with 10 ml of buffered peptone water. The total surface area of each bowl was calculated using the bowl dimensions in order to standardize the bacterial counts per cm^2^. Samples were maintained at 4°C following collection and processed within 24 h.

ISO procedures were used for TMABc and *Enterobacteriaceae* counts (ISO 4833–1:2013 and ISO 21528–2:2017, respectively). Briefly, for the enumeration of TMAB, samples were diluted in buffered peptone water (BPW; CM 509 B, Oxoid) and plated onto Plate Count Agar (PCA CM 0325 Oxoid), then incubated at 31°C for 48 h. For the enumeration of *Enterobacteriaceae*, Violet Red Bile Glucose Agar (VRBG agar CM 0485 Oxoid, Rodano, Milan) was streaked and incubated at 37°C for 48 h. The results are expressed in CFU/cm^2^. The isolation of *Salmonella* spp. was carried out in accordance with ISO 6579–1:2017. After pre-enrichment in BPW for 24 h at 37°C, 1 and 0.1 ml of each pre-enrichment solution was inoculated into 10 ml of Muller Kauffmann Tetrathionate Novobiocin Broth (CM 0343, Oxoid) and 10 ml of Rappaport–Vassiliadis Broth (CM 669 B, Oxoid), respectively, and then incubated at either 37°C (Muller Kauffmann Tetrathionate Novobiocin Broth) or 41°C (Rappaport–Vassiliadis Broth) for 24 h and plated onto selective Xylose Lysine Deoxycholate (XLD) Agar (CM 0469, Oxoid) and Brilliant Green Agar (BGA) (CM 0263, Oxoid). Following 24 h incubation, suspect colonies of *Salmonella* spp. were tested by inoculation into Kligler iron agar (CM0033, Oxoid).

The isolation of Verotoxigenic *E. coli* (VTEC) was performed in accordance with ISO 16649–12:2001 using tryptone bile x-glucuronide (TBX) medium (Oxoid Ltd). Plates were incubated at 41°C per 24 h. In case of presumptive colonies, confirmation was done according to Bottero et al. [22]. The isolation of *Campylobacter* spp. was performed as described in ISO 16140-AOAC using CampyFood broth and agar (Biomerieux). Plates were incubated at 41°C in a microaerophilic environment for 48 h and suspect colonies were tested by Microflex LT MALDI Biotyper mass spectrometer (Bruker Daltonics).

### Statistics

Summary statistics of the survey are reported as totals and proportional percentages. Statistical analyses were performed on the data obtained from the microbiological contamination of 96 dog food bowls. To proceed with statistics, dog food bowls were divided according to:

- (1) Food type (FT) used
  -wet food includes all feed with a moisture level > 14%, whether it be industrial or homemade (48 bowls)
  -dry food includes feed with a moisture level ≤ 14% (i.e., kibble) (48 bowls)
- (2) Cleaning method (CM), divided according to the methods used to remove contamination, organic material and debris
  -dry wiping involves the use of mechanical methods like wiping or brushing but no use of water (51 bowls)
  -hand washing involves water with sponge in the presence or absence of detergent (40 bowls)
  -dishwasher involves the washing of food bowls using a dishwasher (5 bowls)
- (3) Bowl material (BM)
  -plastic (48 bowls)
  -metal (stainless steel or similar, 48 bowls)

Data were analysed using JMPpro v17 software (SAS Institute). For log-scale analyses, all raw values were increased by 0.01 to allow the base 10 logarithm (log_10_) transformation to be applied to the 0 values [1]. A linear mixed model was assessed to study the differences in microbiological contamination between hand washing *vs*. dishwasher. A second, similar model was assessed using the FT (wet food *vs*. dry food), CM (hand washing *vs*. dry wiping), BM (plastic *vs*. metal) and their interactions as the main fixed effects. Each sampled bowl was considered an experimental unit. The results are expressed numerically as mean values and standard error of mean (SEM) and presented graphically as pie and box plots. A *p*-value ≤ 0.05 was considered significant.

## Data Availability

The datasets generated and/or analysed during the current study are not publicly available due to the fact that they are part of a larger ongoing research project with additional data that will be published in the future, but they are available from the corresponding author on reasonable request.

## Abbreviations

FT: Food type
CM: Cleaning method
BM: Bowl material
TMABc: Total mesophilic aerobic bacterial counts
